# Exposure to violence and risk of hypertensive disorders in pregnancy: Systematic review and meta-analysis

**DOI:** 10.1016/j.eurox.2025.100398

**Published:** 2025-05-17

**Authors:** Prakasini Satapathy, Abhay M. Gaidhane, Nasir Vadia, Soumya V. Menon, Kattela Chennakesavulu, Rajashree Panigrahi, Ganesh Bushi, Mahendra Singh, Sanjit Sah, Awakash Turkar, S. Govinda Rao, Khang Wen Goh, Muhammed Shabil, Edward Mawejje

**Affiliations:** a Center for Global Health Research, Saveetha Medical College and Hospital, Saveetha Institute of Medical and Technical Sciences, Saveetha University, Chennai, India; b Faculty of Data Science and Information Technology, INTI International University, Nilai, Malaysia; c Jawaharlal Nehru Medical College, and Global Health Academy, School of Epidemiology and Public Health. Datta Meghe Institute of Higher Education, Wardha, India; d Marwadi University Research Center, Department of Pharmaceutical Sciences, Faculty of Health Sciences, Marwadi University, Rajkot, Gujarat 360003, India; e Department of Chemistry and Biochemistry, School of Sciences, JAIN (Deemed to be University), Bangalore, Karnataka, India; f Department of Chemistry, Sathyabama Institute of Science and Technology, Chennai, Tamil Nadu, India; g Department of Microbiology, IMS and SUM Hospital, Siksha 'O' Anusandhan (Deemed to be University), Bhubaneswar, Odisha 751003, India; h School of Pharmaceutical Sciences, Lovely Professional University, Phagwara, India; i Department of Biotechnology, Graphic Era (Deemed to be University), Clement Town, Dehradun 248002, India; j Graphic Era Hill University, Clement Town, Dehradun, India; k Department of Paediatrics, Dr. D. Y. Patil Medical College Hospital and Research Centre, Dr. D. Y. Patil Vidyapeeth (Deemed-to-be-University), Pimpri, Pune, Maharashtra 411018, India; l Department of Public Health Dentistry, Dr. D.Y. Patil Dental College and Hospital, Dr. D.Y. Patil Vidyapeeth (Deemed-to-be-University), Pune, Maharashtra 411018, India; m Department of Medicine, Korea Universtiy, Seoul, South Korea; n Centre for Research Impact and Outcome, Chitkara University Institute of Engineering and Technology, Chitkara University, Rajpura, Punjab 140401, India; o Division of Research and Innovation, Uttaranchal University, Dehradun, India; p Department of Data Science, Gokaraju Rangaraju Institute of Engineering and Technology, Bachupally, Hyderabad, Telangana 500090, India; q Faculty of Mathematics and Natural Sciences, Universitas Negeri Padang, Padang, Indonesia; r Global Center for Evidence Synthesis, Chandigarh, 160036, India; s Medical Laboratories Techniques Department, AL-Mustaqbal University, Hillah, Babil 51001, Iraq; t School of Public Health, Makerere University College of Health Sciences, Mulago Hill, Kampala, Uganda

**Keywords:** Hypertensive disorders in pregnancy, Violence, Intimate partner violence, Preeclampsia, Gestational hypertension

## Abstract

**Background:**

Hypertensive disorders during pregnancy (HDP) are a significant global health concern, affecting 5–10 % of pregnancies and contributing to maternal and newborn morbidity and mortality. While various factors, including genetics and physiological changes, influence the development of HDP, emerging evidence suggests a strong association with psychosocial stressors, particularly exposure to violence. This meta-analysis aimed to assess the association between exposure to violence and risk of hypertensive disorders in pregnancy.

**Methods:**

A literature review was performed across EMBASE, PubMed, and Web of Science from their inception until October 2024. The review included observational studies that examined exposure to various type of violence and their association with hypertensive disorders in pregnancy. Pooled effect estimates, including odds ratios (ORs) and relative risks (RRs) were computed using a random-effects model.

**Results:**

Sixteen studies met the inclusion criteria. Meta-analysis revealed that violence exposure during pregnancy was associated with a significantly increased risk of HDP, with a pooled OR of 1.380 (95 % CI: 1.079–1.765) and a pooled RR of 1.235 (95 % CI: 1.074–1.420). Subgroup analysis indicated that cohort studies demonstrated a stronger association (OR: 1.726, 95 % CI: 1.182–2.519) compared to cross-sectional studies (OR: 1.112, 95 % CI: 1.009–1.226).

**Conclusion:**

Experiencing violence during pregnancy is significantly linked to a heightened risk of HDP, indicating the need for regular violence screening and early preventive measures in prenatal care. Public health initiatives focused on reducing violence against women, especially during pregnancy, are crucial for improving maternal health and lowering the incidence of hypertensive complications.

## 1 Introduction

Hypertensive disorders in pregnancy (HDP) represent a major global public health issue, playing a significant role in maternal and neonatal illness and death [1]. These conditions, which encompass chronic hypertension, gestational hypertension, preeclampsia, and eclampsia, affect around 5–10 % of pregnancies worldwide and are linked to negative outcomes such as preterm birth, intrauterine growth restriction, and stillbirth [2]. While the causes of hypertensive disorders in pregnancy are multifaceted, involving a complex interaction of genetic, physiological, and environmental factors, growing evidence indicates that psychosocial stressors, especially exposure to violence, may significantly contribute to the increased risk of developing HDP [3]. Grasping the connection between violence and hypertensive disorders in pregnancy is crucial for crafting effective prevention strategies and enhancing maternal health outcomes.

Violence in its various forms, whether physical, sexual or Intimate partner violence (IPV) remains a pervasive issue affecting women globally, with pregnant women being particularly vulnerable. IPV is among the most prevalent forms of violence encountered by women during pregnancy [4]. The World Health Organization (WHO) estimates that 30 % of women globally have experienced physical or sexual violence from an intimate partner at some point in their lives [5]. In the context of pregnancy, this figure can be even higher, with studies reporting rates of IPV ranging from 1 % to over 20 % depending on the geographic and cultural context [6]. Additionally, exposure to community violence, war, and other forms of trauma have also been implicated in adverse pregnancy outcomes. The stress associated with violence exposure may contribute to the development of hypertensive disorders through several mechanisms, including neuroendocrine dysregulation, inflammatory pathways, and alterations in cardiovascular function [7].

The physiological response to stress, particularly chronic stress as a result of violence exposure, is primarily mediated by the hypothalamic-pituitary-adrenal (HPA) axis and the sympathetic nervous system are activated in response to stress, triggering the release of stress hormones like cortisol and catecholamines [8], which can contribute to vascular dysfunction, endothelial injury, and increased blood pressure [9]. Additionally, women who experience multiple forms of violence may be at an even higher risk, as the cumulative impact of these stressors could exacerbate the physiological effects on the cardiovascular system. In pregnancy, the maternal cardiovascular system undergoes significant changes to accommodate the growing fetus, including increases in blood volume and cardiac output, as well as decreased vascular resistance [10]. Chronic stress, however, may disrupt these adaptive changes, increasing the risk of hypertensive disorders. Furthermore, exposure to violence may lead to behavioral changes such as increased smoking, alcohol use, and poor nutrition, which are recognized risk factors for a variety of health conditions, including HDP [11].

A growing body of literature has explored the association between violence exposure and adverse maternal outcomes, including hypertensive disorders. Numerous studies have shown a positive association between IPV and the onset of preeclampsia and gestational hypertension [12], [13]. However, the findings have not been consistent across all studies, with some failing to show a significant association or reporting varied effects based on the type or severity of violence [14]. The inconsistencies in findings could be attributed to variations in study design, population characteristics, and methods used to assess violence, emphasizing the need for a more thorough understanding of the connection between violence and HDP. The implications of this relationship between violence and hypertensive disorders in pregnancy extend beyond maternal health. Hypertensive disorders are a major cause of preterm birth and low birth weight, which can have lasting effects on the child's health and development [15]. Moreover, maternal complications related to HDP, such as eclampsia, can result in severe maternal morbidity and mortality, especially true in low-resource settings, where access to prenatal care and treatment may be limited. Identifying modifiable risk factors, such as exposure to violence, presents a valuable opportunity for intervention to enhance both maternal and neonatal outcomes.

A systematic review and meta-analysis that compiles the existing evidence on the relationship between violence exposure and the risk of HDP can help bridge these knowledge gaps. This type of review can offer more accurate estimates of the strength of this association and highlight areas where additional research is required.

## 2 Method

### 2.1 Study design

This systematic review and meta-analysis was conducted in accordance with the Preferred Reporting Items for Systematic Reviews and Meta-Analyses (PRISMA) guidelines [16], as outlined in Table S1. To ensure transparency and mitigate bias, the protocol for this review was registered in the PROSPERO database.

### 2.2 Search strategy

A comprehensive search was performed across electronic databases such as PubMed, Embase, and Web of Science. The search spanned from the inception of each database up to 10 October 2024, with no limitations on language or publication date. A variety of keyword combinations were employed, utilizing full-text fields to optimize the identification of pertinent studies. The search terms included ("preeclampsia" OR "gestational hypertension") AND ("intimate partner violence" OR "domestic violence"). The detailed search strategy for each database can be found in Table S2.

### 2.3 Eligibility criteria

Studies qualified for inclusion if they fulfilled the following criteria: they adhered to an observational study design (cross-sectional, case-control, or cohort studies); they examined pregnant women subjected to any form of violence including physical, psychological, sexual abuse, or IPV; they documented outcomes pertaining to hypertensive disorders in pregnancy, such as chronic hypertension, gestational hypertension, preeclampsia, or eclampsia; and they provided adequate data to derive effect estimates. Exclusion criteria encompassed studies that were randomized controlled trials, focused on non-pregnant populations, or lacked sufficient data to be incorporated into the meta-analysis.

### 2.4 Study selection

The process of study selection included two steps. In order to find possibly pertinent papers, two independent reviewers first went through the titles and abstracts of every reference that was retrieved. Disagreements were settled by conversation or by seeking advice from a third reviewer. Full-text papers were examined in the second phase in accordance with the established eligibility requirements. The reviewers reached a consensus to settle any disagreements during this stage. To aid in the study selection process, we utilized semi-automated software (Nested-Knowledge, MN, USA) for deduplication and screening of the studies.

### 2.5 Data extraction and risk of bias assessment

Two independent reviewers used a standardized data extraction form to obtain data from the included studies. Study characteristics (e.g., author, year, location, study design), population information (e.g., sample size, age), information regarding the types of violence pregnant women were exposed to (e.g., sexual, IPV, domestic violence), and outcomes pertaining to hypertensive disorders were among the information that was retrieved. Relative risks (RRs) and adjusted impact estimates odd ratios (ORs) were retrieved, along with their 95 % CIs; models that accounted for the greatest number of confounding factors were preferred. A third reviewer was consulted or discussed in order to settle any discrepancies in the data extraction. The Newcastle-Ottawa Scale (NOS) for observational studies was used to evaluate the risk of bias for each included study, and the results are shown in Table S3.

### 2.6 Data synthesis and statistical analysis

Statistical software R version 4.4 was used to aggregate the findings from the included studies in a meta-analysis. To account for study variability, a random-effects model was used to aggregate the effect estimates (ORs and RRs) and their 95 % CIs. The I^2^ statistic was used to measure study heterogeneity; values more than 50 % indicated moderate to significant heterogeneity. By visually examining funnel plots and, when necessary, applying Egger's regression test, the existence of publication bias was evaluated. P-values below 0.05 were regarded as statistically significant.

### 2.7 Subgroup analysis

To investigate possible variations in the relationship between exposure to violence and HDP, a subgroup analysis was carried out based on the research design. In particular, the study contrasted findings from cohort and cross-sectional investigations.

## 3 Results

### 3.1 Literature search

**Fig. 1:**
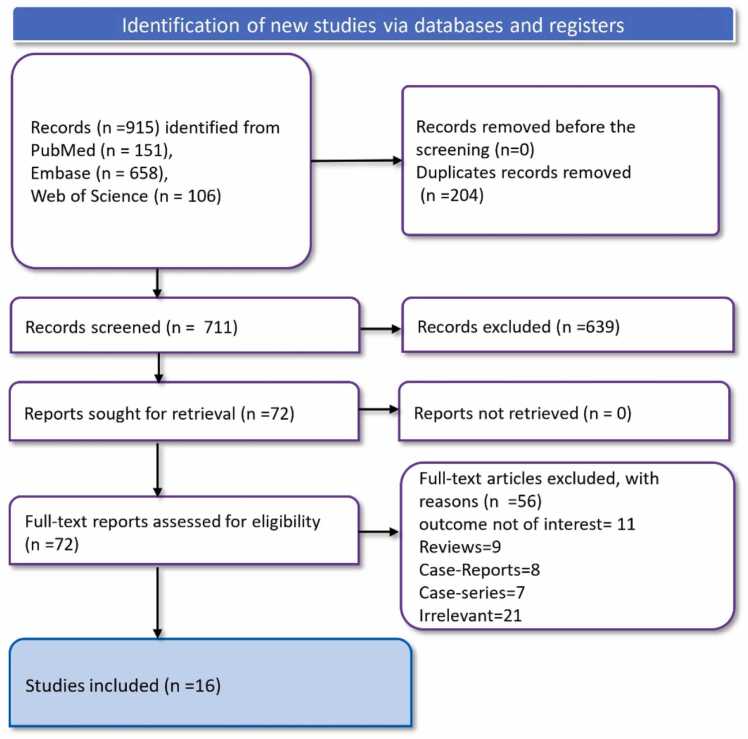
PRISMA flowchart depicting article selection and screening process.

Searches in Web of Science (n = 106), PubMed (n = 151), and Embase (n = 658) yielded a total of 915 studies. 711 articles were left for title and abstract screening after 204 duplicate data were eliminated. After this preliminary analysis, 72 full-text publications remained for additional assessment after 639 research were eliminated. 56 of these were disqualified for a variety of reasons, including irrelevant studies [21] and case reports [8], case series [7], review articles [9], and irrelevant results [11]. In the end, 16 [17], [18], [19], [20], [21], [22], [23], [24], [25], [26], [27], [28], [29], [30], [31], [32] Articles that satisfied the requirements were added to the final analysis (Fig. 1).

### 3.2 Characteristics of included studies

The 16 studies included in this review encompassed a range of geographic locations, study designs, and population characteristics. Most of the research was carried out in wealthy nations like the United States, Canada, and European nations, while a few studies were conducted in middle- or low-income settings, such as South Africa, Jordan, Uganda, and Pakistan. The included studies comprised both cohort and cross-sectional designs, with sample sizes ranging from small-scale studies involving less than 100 participants to large-scale studies with over 300,000 participants. The types of violence assessed varied, including IPV, domestic violence, physical and sexual. Violence was either self-reported or recorded using structured questionnaires such as the Domestic Violence Questionnaire Screening Tool (DVQST) or coded via The International Classification of Diseases (ICD) system is used in medical records. A thorough synopsis of the features of the included studies is given in Table 1.

**Table 1:** Characteristics of included studies.

**Study**	**Country**	**Study design**	**Population characteristics**	**Sample size (violence)**	**Sample size (control)**	**Age**	**Type of violence**	**Effect size (95 % CI for hypertension)**	**Diagnostic criteria (violence)**
Abrahams [17]	South Africa	Cohort	Women who reported violence during pregnancy	209	244	16–40	Rape	OR= 1.77 (1.02–3.08)	Self-reported data
Abujilban [18]	Jordan	Cross-sectional	Married women with singleton pregnancy	67	156	27 (SD = 5.7)	Physical Violence	OR= 4.1 (1.3–13)	Arabic WHO’s (DVQST)
Arcos [19]	Chile	Cohort	Pregnant women who experienced DV	NA	NA	NA	DV	RR= 1.5 (1.18–1.96)	NA
Auger [20]	Canada	Retrospective cohort	Pregnant Women Hospitalized for Physical Assault, Sexual Assault, and IPV	3230	NA	21.5 (SD=7.2)	Physical or Sexual Assault or Intimate Partner Violence	(RR); Physical= 1.08 (0.85–1.37), Sexual= 1.07 (0.61–1.89), IPV= 1.15 (0.87–1.50)	ICD
Berenson [21]	USA	Cohort	Women who reported being physically abused during pregnancy	32	352	21.8 (SD=5.3)	Physically assaulted	OR= 1.1 (0.3–3.3)	Questions included whether the patient had ever been slapped, kicked, hit, or otherwise physically hurt
De Pins [22]	USA	Cross-sectional	Women exposed to IPV during pregnancy	2762	NA	NA	IPV	OR= 1.13 (1.02–1.26)	NA
Garabedian [23]	USA	Cohort	Women exposed to lifetime violence	65	NA	NA	NA	RR= 2.0 (1.2–3.4)	NA
Greely [24]	USA	Cross-sectional	Pregnancy related hospitalizations of women	3,05,145	13,83,11,788	35–49	IPV	OR= 11.95 (7.34–17.82)	ICD−9
Hartwell [25]	USA	Cross-sectional	Pregnant individuals reporting having experienced IPV	5482	1,35,335	25–29	IPV	OR= 1.21 (1.02–1.44)	PRAMS question
Hayer [27]	USA	Retrospective cohort	Women had reported violence in pregnancy	3457	17,25,033	18–35	NA	RR= 1.34 (1.11–1.61)	ICD−10
Hayer [26]	USA	Retrospective cohort	Women with IPV in pregnancy	2756	NA	NA	IPV	OR= 1.41 (1.21–1.65)	NA
Kaye [28]	Uganda	Prospective cohort	Women recruited in the second pregnancy trimester exposed to DV	169	443	NA	DV	OR= 1.40 (1.06–1.85)	Abuse Assessment Screen
Khatoon [29]	India	Cohort	Pregnant women reporting to the outpatient department exposed to DV	60	270	24.5 (SD= 3.3)	DV	RR= 1.40 (0.57–3.45)	WHO questionnaire
Lin [30]	Taiwan	Retrospective cohort	Women who were pregnant for their first childbirth	1322	4,85,981	NA	DV	OR= 2.94 (2.58–3.34)	NA
Silverman [31]	USA	Cross-sectional	Pregnant women that experienced DV	1304	NA	18 or above	IPV	OR= 0.94 (0.74–1.18)	Survey questions from PRAMS
Yasmin 2021 [32]	Pakistan	Cross-sectional	Patients were recruited in third trimester of pregnancy exposed to DV	55	49	NA	DV	OR= 1.423 (0.844–2.398)	NA

**Abbreviations:** PIH: Pregnancy-Induced Hypertension, OR: Odds Ratio, RR: Relative Risk, IPV: Intimate Partner Violence, DV: Domestic Violence, DVQST: Domestic Violence Questionnaire Screening Tool, SD: Standard Deviation, PRAMS: Pregnancy Risk Assessment Monitoring System, ICD: International Classification of Diseases, WHO: World Health Organization

### 3.3 Association between violence exposure and risk of HDP

**Fig. 2:**
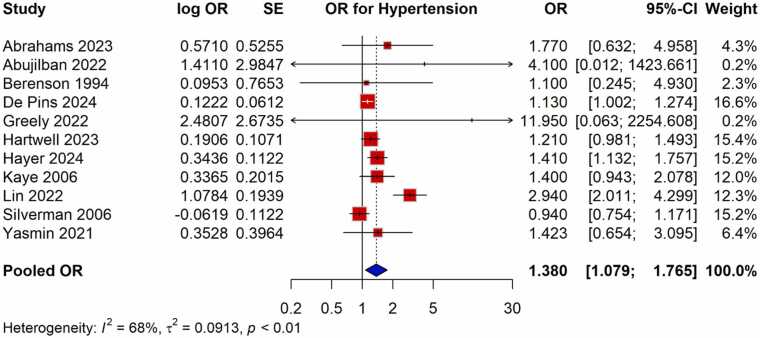
Meta-analysis of pooled OR showing association between violence exposure and the risk of HDP.

**Fig. 3:**
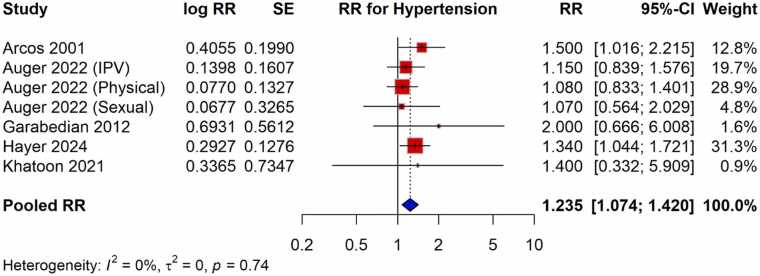
Meta-analysis of pooled RR showing association between violence exposure and the risk of HDP.

The meta-analysis found a strong positive correlation between the risk of HDP and exposure to violence during pregnancy. There was a 38 % higher chance of developing HDP for pregnant women who experienced violence than for those who did not, according to the pooled OR from studies evaluating the relationship between violence and HDP, which was 1.380 (95 % CI: 1.079–1.765) (Fig. 2). The moderate heterogeneity across trials (I^2^ = 68 %) led to the choice of the random-effects model. With a pooled RR of 1.235 (95 % CI: 1.074–1.420), studies that published RR measures similarly showed a significant correlation, indicating a comparable heightened risk (Fig. 3). Exposure to violence and hypertension outcomes during pregnancy are consistently linked, according to the findings of both OR and RR meta-analyses.

### 3.4 Subgroup analysis

**Fig. 4:**
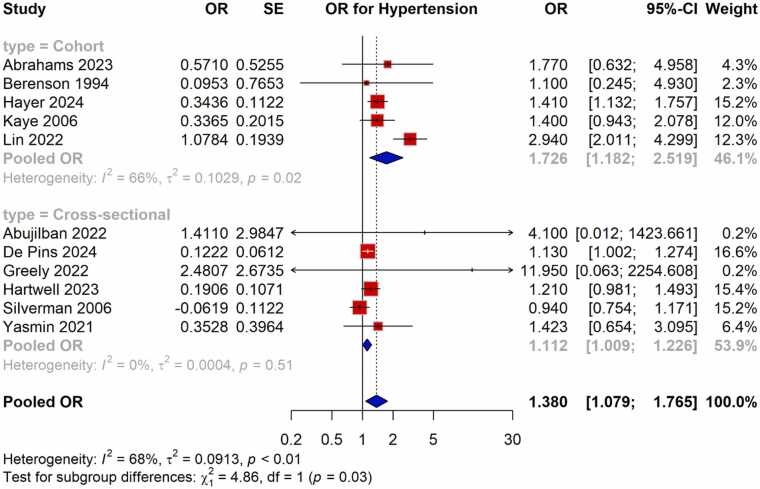
Subgroup analysis of pooled OR showing the association between violence exposure and the risk of HDP.

To investigate possible causes of heterogeneity, subgroup analyses were carried out, with a special emphasis on the research design type (cohort vs. cross-sectional). Cohort studies had a pooled OR of 1.726 (95 % CI: 1.182–2.519, I² = 66 %), whereas cross-sectional studies had a pooled OR of 1.112 (95 % CI: 1.009–1.226, I² = 0 %). The statistical significance of the test for subgroup differences (p = 0.03) suggests that the study design played a role in the overall analysis's heterogeneity (Fig. 4).

### 3.5 Sensitivity analysis

**Fig. 5:**
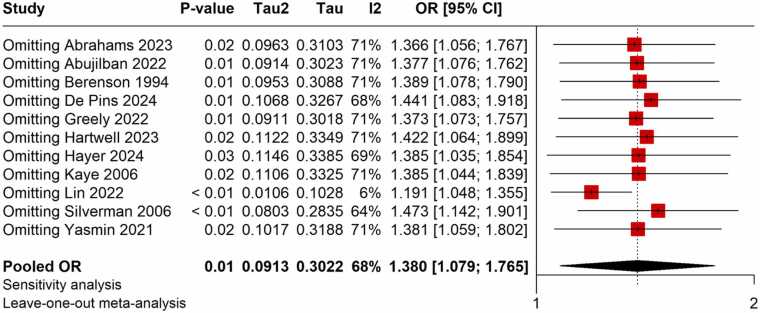
Sensitivity analysis of pooled OR showing the association between violence exposure and the risk of HDP.

**Fig. 6:**
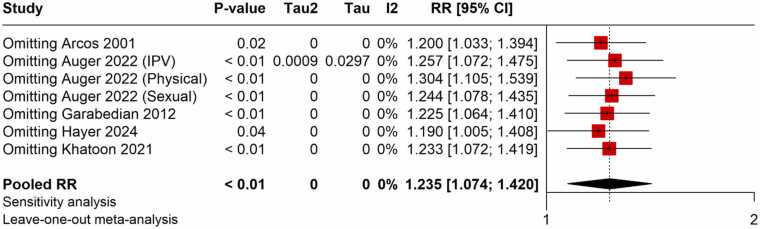
Sensitivity analysis of pooled RR showing the association between violence exposure and the risk of HDP.

To gauge the integrity of the findings, a leave-one-out sensitivity analysis was conducted. According to this analysis, the overall pooled impact estimate was largely consistent across different individual study exclusions, with RRs ranging from 1.190 to 1.304 (Fig. 6) and ORs ranging from 1.191 to 1.473 (Fig. 5). The dependability of the link between exposure to violence and hypertensive problems during pregnancy was confirmed by the fact that none of the excluded studies significantly affected the overall findings.

### 3.6 Publication bias

**Fig. 7:**
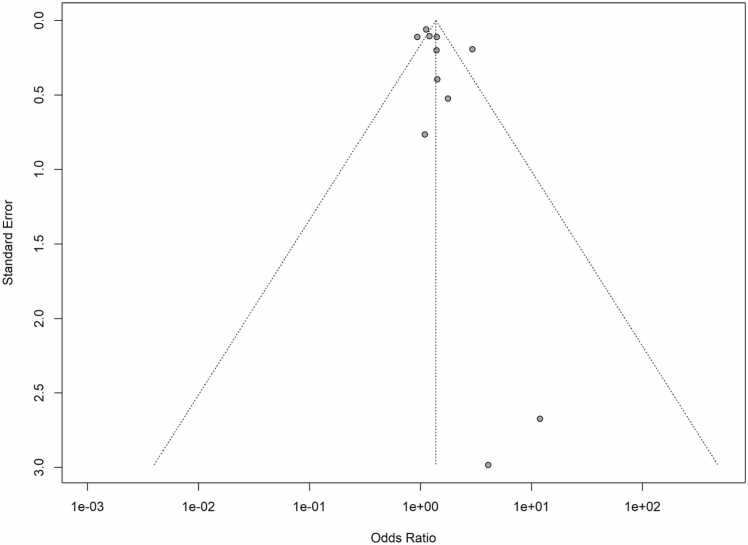
Funnel plot.

Egger's regression test and a funnel plot (Fig. 7) were used to evaluate publication bias. With the majority of the research grouped at the top of the funnel plot, which seems symmetrical, there isn't much indication of publication bias. At the bottom of the figure, smaller studies with bigger standard errors are more widely distributed. With a p-value of 0.1917, Egger's regression test provided additional evidence that there was no substantial publication bias. This p-value suggests that there is no statistically significant evidence of publication bias in the included research because it is higher than the generally accepted cut-off of 0.05.

## 4 Discussion

The findings of this meta-analysis and systematic review provide strong evidence supporting a significant association between prenatal exposure to violence and the risk of HDP. Based on the pooled ORs and RRs from the included studies, women who have experienced physical, sexual, IPV, or rape are 38 % and 23 % respectively, to develop HDP than those who have not. The consistency of these findings across both OR and RR measures, regardless of the statistical approach, reinforces the validity of our conclusions and indicates that women exposed to violence have a higher likelihood of developing HDP. The agreement between OR and RR estimates further strengthens the claim that violence is a significant risk factor for hypertensive complications during pregnancy. This outcome aligns with the growing body of research recognizing psychological stressors as major contributors to adverse pregnancy outcomes.

Previous studies have examined the relationship between exposure to violence and unfavorable pregnancy outcomes, such as HDP, but the results have been inconsistent in terms of their importance and intensity. According to earlier meta-analyses, 51.5 % of pregnant women experience intimate partner violence (95 % CI: 45.0–58.1), which might help explain why there is a higher chance of unfavorable pregnancy outcomes such as hypertensive disorders [33]. Furthermore, a recent Umbrella review that evaluated the effect of IPV on maternal outcomes discovered that the preterm birth rate among mothers were 11 % higher for women exposed to IPV, including pregnancy-induced hypertension (POR: 4.13; 95 % CI 3.17–5.10) [34]. Moreover, our analysis incorporated more recent studies and expanded the focus to include not only IPV but also broader categories of violence, such as physical and sexual violence, which could contribute to a stronger association. The findings we obtained are in line with earlier studies, including one that found pregnant women who experienced IPV had a 2.4-fold higher risk of preeclampsia (OR = 2.4; 95 % CI: 1.7–3.3). This emphasizes the significance of early intervention for women at risk and provides more evidence of the strong correlation between exposure to violence and hypertension problems during pregnancy [12].

Several biological and psychosocial mechanisms may explain the observed link between violence exposure and the increased risk of HDP. Chronic stress resulting from violence and adverse psychosocial experiences has been recognized as a major contributor to cardiovascular disease [35], and this pathway is particularly relevant during pregnancy. Prolonged stress triggers the sympathetic nervous system and HPA axis, which raises levels of stress hormones including cortisol and catecholamines. These substances can result in inflammation, endothelial dysfunction, and raised blood pressure [8]. During pregnancy, the maternal cardiovascular system undergoes extensive physiological changes, including increased blood volume and cardiac output. These changes make pregnant women particularly vulnerable to stress-induced hypertension [36]. Moreover, violence may indirectly contribute to HDP through behavioral pathways. Unhealthy coping mechanisms including smoking, drinking, and eating poorly are all known risk factors for HDP, and they are especially common among women who experience abuse [37]. The interaction between these behavioral risk factors and the physiological stress response could further exacerbate the risk of developing hypertensive complications during pregnancy. Given these complex interactions, it is critical that healthcare providers adopt a multidisciplinary approach to managing pregnant women exposed to violence, addressing both the psychological and physical health needs of this vulnerable population.

Our subgroup analysis revealed notable differences in the strength of the association between violence exposure and HDP based on study design. Specifically, cohort studies demonstrated a stronger pooled OR compared to cross-sectional studies. The longitudinal nature of cohort studies allows for a more accurate assessment of the temporal relationship between exposure and outcome, which may explain the stronger association observed in these studies. Cross-sectional studies, on the other hand, provide only a snapshot of the exposure and outcome at one point in time, limiting the ability to infer causality. The heterogeneity observed between studies (I² = 68 %) suggests that the relationship between violence and HDP may vary depending on factors such as population characteristics, the type of violence assessed, and the method of violence measurement. For example, studies conducted in low- and middle-income countries (LMICs) may report higher ORs due to the compounding effects of poverty, lack of healthcare access, and cultural norms that may exacerbate the impact of violence on maternal health. Additionally, the way violence is measured, whether through self-reported questionnaires or medical records may influence the strength of the observed association, as self-report measures are more susceptible to underreporting due to social stigma or fear of retaliation. The sensitivity analysis using a leave-one-out approach confirmed the robustness of our findings. The overall pooled OR remained stable across the omission of individual studies, with effect estimates ranging from 1.191 to 1.473. Similarly, the pooled RR remained consistent within a narrow range (1.190–1.304). This suggests that no single study disproportionately influenced the overall results, reinforcing the reliability of the observed association between violence exposure and HDP.

The findings of this review have important implications for both clinical practice and public health policy. Given the substantial risk of HDP associated with violence exposure, routine screening for violence during pregnancy should be integrated into prenatal care services. Early identification of women at risk can facilitate timely interventions, such as counseling, social support, and referrals to protective services, which may help mitigate the negative impact of violence on maternal health outcomes. Furthermore, public health programs aimed at preventing violence against women should prioritize pregnant women as a particularly vulnerable group. Community-based interventions that address the root causes of violence, such as gender inequality, poverty, and lack of education, may help reduce the prevalence of violence and, consequently, the incidence of HDP and other adverse pregnancy outcomes. In addition, healthcare providers should be trained to recognize the signs of violence and provide trauma-informed care to pregnant women, ensuring that they receive comprehensive and compassionate support.

This study's thorough and methodical approach to identifying and synthesizing the existing information is one of its main advantages. This meta-analysis offers a more complex picture of the connection between violence and HDP by encompassing a broad range of study designs and forms of violence. Additionally, by using subgroup analysis, we were able to investigate how study design and demographic variables affected the association's strength, which gave us important information for further research. Nonetheless, a number of restrictions must be noted. First, the findings' generalizability is constrained by the variation throughout research, especially with regard to demographic characteristics and violence assessment. Second, while we made efforts to account for confounding factors by prioritizing studies with adjusted effect estimates, residual confounding remains a potential issue in observational studies. Finally, the reliance on self-reported data in some studies may introduce reporting bias, particularly in settings where violence is stigmatized or underreported.

While this review highlights a significant association between violence exposure and HDP, further research is needed to explore the underlying mechanisms driving this relationship. Longitudinal studies with detailed assessments of both psychological and physiological stress markers are needed to disentangle the complex interactions between stress, behavior, and maternal cardiovascular health. Additionally, future studies should explore the impact of interventions aimed at reducing violence exposure on the incidence of HDP and other pregnancy complications.

## 5 Conclusion

A strong correlation between exposure to violence during pregnancy and a higher risk of hypertensive diseases, such as gestational hypertension and preeclampsia, is demonstrated by this comprehensive review and meta-analysis. The results highlight how important it is to regularly screen for violence in prenatal care and incorporate early treatments to lessen its detrimental impact on mother health.

## CRediT authorship contribution statement

**Abhay M Gaidhane:** Data curation, Formal analysis. **Edward Mawejje:** Visualization, Writing – original draft. **Nasir Vadia:** Writing – original draft. **Soumya V Menon:** Investigation, Writing – review & editing. **Kattela Chennakesavulu:** Supervision. **Rajashree Panigrahi:** Formal analysis, Investigation. **Ganesh Bushi:** Writing – original draft, Writing – review & editing. **Mahendra Singh:** Resources, Software. **Awakash Turkar:** Formal analysis, Writing – review & editing. **S. Govinda Rao:** Conceptualization, Formal analysis, Investigation. **Sanjit Sah:** Software, Supervision, Validation. **Khang Wen Goh:** Writing – review & editing. **Prakasini Satapathy:** Conceptualization. **Muhammed Shabil:** Writing – original draft.

## Declaration of Competing Interest

The authors declare that they have no known competing financial interests or personal relationships that could have appeared to influence the work reported in this paper.

## Data Availability

The data is available within the manuscript and supplementary material.

## Footnotes

Appendix A Supplementary data associated with this article can be found in the online version at doi:10.1016/j.eurox.2025.100398.
