# Supplementary material for: Exposure to violence and risk of hypertensive disorders in pregnancy: Systematic review and meta-analysis
Source: Eur J Obstet Gynecol Reprod Biol X. 2025 May 17;26:100398. doi: 10.1016/j.eurox.2025.100398 (PMC12166821; doi:10.1016/j.eurox.2025.100398)
Supplement: Supplementary file 1 — Supplementary material [file mmc1.docx]

**SUPPLEMENTARY MATERIALS**

**Table S1.** PRISMA Checklist

| **Section and Topic** | **Item #** | **Checklist item** | **Location where item is reported** |
| --- | --- | --- | --- |
| **TITLE** | | |  |
| Title | 1 | Identify the report as a systematic review. | 1 |
| **ABSTRACT** | | |  |
| Abstract | 2 | See the PRISMA 2020 for Abstracts checklist. (made as per the Journal guidelines) | 2 |
| **INTRODUCTION** | | |  |
| Rationale | 3 | Describe the rationale for the review in the context of existing knowledge. | 3 |
| Objectives | 4 | Provide an explicit statement of the objective(s) or question(s) the review addresses. | 3 |
| **METHODS** | | |  |
| Eligibility criteria | 5 | Specify the inclusion and exclusion criteria for the review and how studies were grouped for the syntheses. | 3 |
| Information sources | 6 | Specify all databases, registers, websites, organisations, reference lists and other sources searched or consulted to identify studies. Specify the date when each source was last searched or consulted. | 3 |
| Search strategy | 7 | Present the full search strategies for all databases, registers and websites, including any filters and limits used. | Table S2 |
| Selection process | 8 | Specify the methods used to decide whether a study met the inclusion criteria of the review, including how many reviewers screened each record and each report retrieved, whether they worked independently, and if applicable, details of automation tools used in the process. | 4 |
| Data collection process | 9 | Specify the methods used to collect data from reports, including how many reviewers collected data from each report, whether they worked independently, any processes for obtaining or confirming data from study investigators, and if applicable, details of automation tools used in the process. | 4 |
| Data items | 10a | List and define all outcomes for which data were sought. Specify whether all results that were compatible with each outcome domain in each study were sought (e.g., for all measures, time points, analyses), and if not, the methods used to decide which results to collect. | 4, Table 1 |
|  | 10b | List and define all other variables for which data were sought (e.g., participant and intervention characteristics, funding sources). Describe any assumptions made about any missing or unclear information. | 4 |
| Study risk of bias assessment | 11 | Specify the methods used to assess risk of bias in the included studies, including details of the tool(s) used, how many reviewers assessed each study and whether they worked independently, and if applicable, details of automation tools used in the process. | 4, |
| Effect measures | 12 | Specify for each outcome the effect measure(s) (e.g. risk ratio, mean difference) used in the synthesis or presentation of results. | 5 |
| Synthesis methods | 13a | Describe the processes used to decide which studies were eligible for each synthesis (e.g. tabulating the study intervention characteristics and comparing against the planned groups for each synthesis (item #5)). | 5, Table 1 |
|  | 13b | Describe any methods required to prepare the data for presentation or synthesis, such as handling of missing summary statistics, or data conversions. | NA |
|  | 13c | Describe any methods used to tabulate or visually display results of individual studies and syntheses. | 5 |
|  | 13d | Describe any methods used to synthesize results and provide a rationale for the choice(s). If meta-analysis was performed, describe the model(s), method(s) to identify the presence and extent of statistical heterogeneity, and software package(s) used. | 5 |
|  | 13e | Describe any methods used to explore possible causes of heterogeneity among study results (e.g. subgroup analysis, meta-regression). | 5 |
|  | 13f | Describe any sensitivity analyses conducted to assess robustness of the synthesized results. | 5 |
| Reporting bias assessment | 14 | Describe any methods used to assess risk of bias due to missing results in a synthesis (arising from reporting biases). | NA |
| Certainty assessment | 15 | Describe any methods used to assess certainty (or confidence) in the body of evidence for an outcome. | NA |
| **RESULTS** | | |  |
| Study selection | 16a | Describe the results of the search and selection process, from the number of records identified in the search to the number of studies included in the review, ideally using a flow diagram. | 5, Figure-1, 2 |
|  | 16b | Cite studies that might appear to meet the inclusion criteria, but which were excluded, and explain why they were excluded. | 5, Table 1 |
| Study characteristics | 17 | Cite each included study and present its characteristics. | Table-1 |
| Risk of bias in studies | 18 | Present assessments of risk of bias for each included study. | Table S3 |
| Results of individual studies | 19 | For all outcomes, present, for each study: (a) summary statistics for each group (where appropriate) and (b) an effect estimate and its precision (e.g. confidence/credible interval), ideally using structured tables or plots. | Table 1, 3 |
| Results of syntheses | 20a | For each synthesis, briefly summarise the characteristics and risk of bias among contributing studies. | 5 |
|  | 20b | Present results of all statistical syntheses conducted. If meta-analysis was done, present for each the summary estimate and its precision (e.g. confidence/credible interval) and measures of statistical heterogeneity. If comparing groups, describe the direction of the effect. | 5, 6 |
|  | 20c | Present results of all investigations of possible causes of heterogeneity among study results. | 5, 6 |
|  | 20d | Present results of all sensitivity analyses conducted to assess the robustness of the synthesized results. | 7, figure 3,4 |
| Reporting biases | 21 | Present assessments of risk of bias due to missing results (arising from reporting biases) for each synthesis assessed. | NA |
| Certainty of evidence | 22 | Present assessments of certainty (or confidence) in the body of evidence for each outcome assessed. | NA |
| **DISCUSSION** | | |  |
| Discussion | 23a | Provide a general interpretation of the results in the context of other evidence. | 8 |
|  | 23b | Discuss any limitations of the evidence included in the review. | 8 |
|  | 23c | Discuss any limitations of the review processes used. | 8 |
|  | 23d | Discuss implications of the results for practice, policy, and future research. | 9 |
| **OTHER INFORMATION** | | |  |
| Registration and protocol | 24a | Provide registration information for the review, including register name and registration number, or state that the review was not registered. | 4 |
|  | 24b | Indicate where the review protocol can be accessed, or state that a protocol was not prepared. | 4 |
|  | 24c | Describe and explain any amendments to information provided at registration or in the protocol. | NA |
| Support | 25 | Describe sources of financial or non-financial support for the review, and the role of the funders or sponsors in the review. | 10 |
| Competing interests | 26 | Declare any competing interests of review authors. | 10 |
| Availability of data, code and other materials | 27 | Report which of the following are publicly available and where they can be found: template data collection forms; data extracted from included studies; data used for all analyses; analytic code; any other materials used in the review. | 10 |

**Table S2.** The adjusted search terms as per searched electronic databases

| Database | Search Query | Results |
| --- | --- | --- |
|  | | |
| PubMed | ((gestational OR pregnant OR pregnancy OR maternal OR Obstetrics OR obstetric OR "labour" OR "delivery" OR "birth") AND ("hyperten*" OR preeclampsia OR eclampsia OR pre-eclampsia OR "high blood pressure")) AND (("Violence"[All Fields] OR "Domestic violence"[All Fields] OR "Spouse abuse"[All Fields] OR "Elder abuse"[All Fields] OR "Intimate partner violence"[All Fields] OR "Battered women"[All Fields] OR "Gender based violence"[All Fields] OR "Violence against women"[All Fields] OR "Physical violence"[All Fields] OR "Emotional violence"[All Fields] OR "Sexual violence"[All Fields] OR "Emotional violence"[All Fields])) | **151** |
|  | | |
| Embase | ('gestational' OR 'pregnant' OR 'pregnancy'/exp OR 'pregnancy' OR 'maternal'/exp OR 'maternal' OR 'obstetrics'/exp OR 'obstetrics' OR 'obstetric' OR 'labour'/exp OR 'labour' OR 'delivery'/exp OR 'delivery' OR 'birth'/exp OR 'birth') AND ('hypertension'/exp OR 'hypertension' OR 'preeclampsia'/exp OR 'preeclampsia' OR 'eclampsia'/exp OR 'eclampsia' OR 'pre-eclampsia'/exp OR 'pre-eclampsia' OR 'high blood pressure'/exp OR 'high blood pressure') AND ('violence'/exp OR 'violence' OR 'domestic violence'/exp OR 'domestic violence' OR 'spouse abuse'/exp OR 'spouse abuse' OR 'elder abuse'/exp OR 'elder abuse' OR 'intimate partner violence'/exp OR 'intimate partner violence' OR 'battered women'/exp OR 'battered women' OR 'gender-based violence'/exp OR 'gender-based violence' OR 'violence against women'/exp OR 'violence against women' OR 'physical violence'/exp OR 'physical violence' OR 'emotional violence'/exp OR 'emotional violence' OR 'sexual violence'/exp OR 'sexual violence') | 665 |
|  | | |
| Web of Science | ((gestational OR pregnant OR pregnancy OR maternal OR obstetrics OR obstetric OR "labour" OR "delivery" OR "birth") AND ("hypertension" OR preeclampsia OR eclampsia OR pre-eclampsia OR "high blood pressure")) AND (("violence" OR "domestic violence" OR "spouse abuse" OR "elder abuse" OR "intimate partner violence" OR "battered women" OR "gender based violence" OR "violence against women" OR "physical violence" OR "emotional violence" OR "sexual violence" OR "emotional violence")) | 107 |

**Table S3.** Quality assessment using Newcastle-Ottawa Scale

| **STUDY** | **SELECTION (max 4 points)** | | | | **COMPARABILITY (max 2 points)** | **OUTCOME (max 3 points)** | | | **SCORE (out of 9)** |
| --- | --- | --- | --- | --- | --- | --- | --- | --- | --- |
|  | Representativeness | Selection | Ascertainment | Demonstration of the outcome of interest was not present at start of study | Comparability the basis of the design or analysis | Assessment of outcome | Was follow-up long enough for outcomes to occur? | Adequacy of the follow-up |  |
| Abrahams 2023 (1) | 1 | 1 | 1 | 1 | 2 | 1 | 1 | 1 | 9 |
| Abujilban 2022 (2) | 1 | 1 | 1 | NA | 1 | 1 | NA | NA | 5 |
| Arcos 2001 (3) | 0 | 1 | 1 | 1 | 1 | 1 | 1 | 1 | 7 |
| Auger 2022 (4) | 1 | 1 | 1 | 1 | 1 | 1 | 0 | 0 | 6 |
| Berenson 1994 (5) | 1 | 1 | 1 | 1 | 2 | 1 | 0 | 0 | 7 |
| De Pins 2024 (6) | 1 | 1 | 1 | NA | 1 | 1 | NA | NA | 5 |
| Garabedian 2012 (7) | 1 | 1 | 1 | 1 | 1 | 1 | 1 | 1 | 8 |
| Greely 2022 (8) | 1 | 1 | 1 | NA | 1 | 1 | NA | NA | 5 |
| Hartwell 2023 (9) | 1 | 1 | 1 | NA | 1 | 1 | NA | NA | 5 |
| Hayer 2024 (10) | 1 | 1 | 1 | 1 | 2 | 1 | 1 | 1 | 9 |
| Hayer 2024 (11) | 0 | 1 | 1 | 1 | 2 | 1 | 1 | 1 | 8 |
| Kaye 2006 (12) | 1 | 1 | 1 | 1 | 1 | 1 | 1 | 1 | 8 |
| Khatoon 2021 (13) | 1 | 1 | 1 | 1 | 2 | 1 | 1 | 1 | 9 |
| Lin 2022 (14) | 1 | 1 | 1 | 1 | 1 | 1 | 1 | 1 | 8 |
| Silverman 2006 (15) | 1 | 1 | 1 | NA | 1 | 1 | NA | NA | 5 |
| Yasmin 2021 (16) | 1 | 1 | 1 | NA | 1 | 1 | NA | NA | 5 |

**Bibliography**

1. Abrahams N, Chirwa E, Mhlongo S, Seedat S, Myers B, Peer N, et al. Pathways to adverse pregnancy outcomes: exploring the mediating role of intimate partner violence and depression: results from a South African rape cohort study. Arch Womens Ment Health. 2023;26(3).

2. Abujilban S, Mrayan L, Al-Modallal H, Damra JK. Physical Intimate Partner Violence and Maternal Outcomes in a Hospital-Based Sample of Pregnant Women in Jordan. Florence Nightingale J Nurs. 2022;30(3).

3. Arcos E, Uarac M, Molina I, Repossi A, Ulloa M. [Impact of domestic violence on reproductive and neonatal health]. Rev Med Chil. 2001;129(12).

4. Auger N, Low N, Lee GE, Ayoub A, Luu TM. Pregnancy Outcomes of Women Hospitalized for Physical Assault, Sexual Assault, and Intimate Partner Violence. J Interpers Violence. 2022;37(13-14).

5. Berenson AB, Wiemann CM, Wilkinson GS, Jones WA, Anderson GD. Perinatal morbidity associated with violence experienced by pregnant women. Am J Obstet Gynecol. 1994;170(6).

6. De Pins AM, DeBolt CA. Evaluating the Effect of Intimate Partner Violence on Hypertensive Disorders of Pregnancy. Obstet Gynecol. 2024;143(5).

7. Garabedian M, Williams C, Strong M, Garcia L, Hansen W, Coker A. Violence against women increases the risk of gestational hypertensive disorders. Am J Obstet Gynecol. 2012;206(1).

8. Greely JT, Dongarwar D, Crear L, Adeyeye M, Reyna-Carrillo A, Salihu HM. Violence against pregnant women and adverse maternal/ fetal outcomes in the United States: racial/ethnic disparities. Women Health. 2022;62(6).

9. Hartwell M, Keener A, Robling K, Enmeier M, B. Sajjadi N, Greiner B, Price J. Associations of intimate partner violence and maternal comorbidities: a cross-sectional analysis of the Pregnancy Risk Assessment Monitoring System. J Osteopath Med. 2023;123(8).

10. Hayer S, Mnuk RE, Garg B, Caughey AB. Risk of adverse perinatal outcomes with violence in pregnancy. Arch Gynecol Obstet. 2024.

11. Hayer S, Garg B, Caughey AB. 878 Intimate partner violence is associated with increased adverse perinatal outcomes. Am J Obstet Gynecol. 2024;230(1).

12. Kaye DK, Mirembe FM, Bantebya G, Johansson A, Ekstrom AM. Domestic violence during pregnancy and risk of low birthweight and maternal complications: a prospective cohort study at Mulago Hospital, Uganda. Trop Med Int Health. 2006;11(10).

13. Khatoon F, Fatima M, Zaidi Z, Nishad S, Ahmad A. Domestic Violence During Pregnancy: Evaluating the Impact on Maternal and Perinatal Health-A Pilot Study in Uttar Pradesh. J Obstet Gynaecol India. 2021;71(4).

14. Lin CH, Lin WS, Chang HY, Wu SI. Domestic violence against pregnant women is a potential risk factor for low birthweight in full-term neonates: A population-based retrospective cohort study. PLoS ONE. 2022;17(12 December).

15. Silverman JG, Decker MR, Reed E, Raj A. Intimate partner violence victimization prior to and during pregnancy among women residing in 26 U.S. states: associations with maternal and neonatal health. Am J Obstet Gynecol. 2006;195(1).

16. Yasmin S, Shafqat T, Sabir S, Syed W, Liaqat N. Domestic violence as a risk factor for hypertensive disorders of pregnancy. Med Forum Monthly. 2021;32(3).
